# Correction: Davies et al. An Innovative Machine Learning Approach to Predict the Dietary Fiber Content of Packaged Foods. *Nutrients* 2021, *13*, 3195

**DOI:** 10.3390/nu18091367

**Published:** 2026-04-27

**Authors:** Tazman Davies, Jimmy Chun Yu Louie, Tailane Scapin, Simone Pettigrew, Jason HY Wu, Matti Marklund, Daisy H. Coyle

**Affiliations:** 1 The George Institute for Global Health, Faculty of Medicine, University of New South Wales, Sydney, NSW 2042, Australia; jimmyl@hku.hk (J.C.Y.L.); tailane.scapin@deakin.edu.au (T.S.); spettigrew@georgeinstitute.org.au (S.P.); jwu1@georgeinstitute.org.au (J.H.W.); mmarklund@georgeinstitute.org.au (M.M.); dcoyle@georgeinstitute.org.au (D.H.C.); 2 School of Biological Science, Faculty of Science, The University of Hong Kong, Hong Kong 999077, China; 3 Nutrition in Foodservice Research Centre, Federal University of Santa Catarina, Florianopolis 88040-900, Brazil; 4 Department of Epidemiology, John Hopkins Bloomberg School of Public Health, Baltimore, MD 21205, USA; 5 Department of Public Health and Caring Sciences, Uppsala University, 75122 Uppsala, Sweden

The journal’s Editorial Office and Editorial Board are jointly issuing a resolution and removal of the Journal Notice linked to this article [1], as well as an update to the original publication. Following concerns raised about the integrity of the peer review, the Editorial Office has conducted a post-publication peer review of this article. This process included the recruitment of one new independent reviewer and was supervised by the Editorial Board member and the Editors-in-Chief to ensure full compliance with MDPI’s editorial process (https://www.mdpi.com/editorial_process, accessed on 5 March 2026).

As a result of this process, the Editors-in-Chief and the authors have agreed to update the following aspects of this publication:

Reviewer report 2 was removed from the peer review record, and one new report was uploaded to the peer review record (https://www.mdpi.com/2072-6643/13/9/3195/review_report, accessed on 30 March 2026).

## Text Correction

In the original publication [1], we updated the contents in the Materials and Methods Section. With this update, we clarified that the nutrition data was obtained from nutrition information panels. We also expanded our rationale for the exclusion criteria and the chosen train–test split.

In Section 2.1, the second-to-last sentence was newly added: “All nutrition information was derived from the nutrition information panel.”.

In Section 2.2, in the first paragraph, the second-to-last sentence was updated to: “Additionally, we excluded food and beverage categories that either (i) contributed less than 0.8% of fiber intake in Australia as this cutoff represented the lower tail of the distribution of fiber contribution across categories [30] (e.g., ‘cheese’, ‘cooking oils’, and ‘honey’), or (ii) had less than 25% of products reporting fiber content (e.g., ‘processed meat’) (*n* = 41,338) (Supplementary Table S1).”.

In Section 2.2, the second paragraph, the third sentence was newly added: “Stratifying by brand name allowed us to avoid train-test leakage due to near-duplicate products (e.g., it would be inappropriate to train on “Pringles Original” and test on “Pringles BBQ”, as it is unlikely that a brand would report fiber content on one product and not the other in the real-world); however, it did slightly disrupt the proportion of different food categories between the training and test dataset (i.e., the proportion of all products in the test dataset ranged from 11% for ‘pasta’ and 34% for ‘biscuits’).”.

## Data Availability Statement Updated

A correction has been made to the Data Availability Statement. With this update, we have provided the code used for the machine learning algorithm.

The authors state that the scientific conclusions are unaffected. This correction was approved by the Academic Editor. The original publication has also been updated.
