# Supplementary material for: An Innovative Machine Learning Approach to Predict the Dietary Fiber Content of Packaged Foods
Source: Nutrients. 2021 Sep 14;13(9):3195. doi: 10.3390/nu13093195 (PMC8470168; doi:10.3390/nu13093195)
Supplement: Supplementary file 1 [file nutrients-13-03195-s001.zip › nutrients-1355889-supplementary.pdf]

Supplementary Table S1: Included and excluded food categories

Food group	Food category	*n*	Proportion of products that report fiber content (%)	Proportion of dietary fiber intake (%)
Bread and bakery products	Biscuits	2,246	39	1.8
	Bread	2,007	63	14.5
	Cakes, muffins, and pastries	1,581	29	4.2
Cereal and grain products	Breakfast cereals	1,427	87	10.6
	Cereal and nut-based bars	724	89	0.8
	Couscous	71	64	0.0
	Noodles	285	30	0.2
	Pasta	1,014	55	1.7
	Rice	391	62	0.2
	Other cereal and grain products	834	63	2.2
Confectionery	Chocolate and sweets	3,780	12	0.6
	Jelly	172	34	0.0
	Chewing gum	114	93	0.0
	Cough lollies	30	40	0.0
Convenience foods	Pizza	175	25	0.1
	Pre-prepared salads and sandwiches	542	30	0.2
	Ready meals	1,430	40	9.0
	Soup	973	51	2.7
	Meal kits	48	18	0.2
	Other frozen foods not otherwise specified	24	41	0.2
Dairy	Cheese	2,048	6	0.0
	Cream	178	8	0.0
	Desserts	481	16	0.2
	Ice cream and edible ices	1,259	20	0.0
	Milk	1,516	23	0.5
	Yoghurt and yoghurt drinks	1,594	16	0.2
Edible oils and oil emulsions	Cooking oils	67	11	0.0
	Edible oils	358	8	0.0
	Cooking oil spray	38	7	0.0
	Coconut oil	67	11	0.0
Fish and fish products	Processed fish	1,704	11	0.6
Fruit and vegetables	Fruit	1,796	62	15.0
	Herbs and spices	668	8	0.1
	Jam and marmalades	439	15	0.2
	Nuts and seeds	1,262	47	3.8
	Vegetables	2,280	49	18.8
Meat and meat products	Meat alternatives	332	69	0.1
	Processed meat	3,320	11	5.1

Food group	Food category	*n*	Proportion of products that report fiber content (%)	Proportion of dietary fiber intake (%)
Non-alcoholic beverages	Coffee and tea	791	14	1.2
	Cordials	221	41	0.1
	Electrolyte drinks	78	75	0.1
	Energy drinks	131	30	0.1
	Fruit and vegetable juices	1,647	49	1.9
	Soft drinks	1155	32	0.1
	Waters	502	38	0.1
	Beverage mixes	142	45	0.1
Sauces, dressings, spreads, and dips	Mayonnaise and salad dressings	693	7	0.1
	Sauces	3,263	17	0.6
	Spreads and dips	1,450	18	0.4
Snackfoods	Crisps and snacks	1,825	53	1.2
Sugars, honey, and related products	Honey	301	2	0.0
	Dessert toppings	136	11	0.0
	Syrup	149	13	0.0
	Dessert additions	203	23	0.0
	Condensed caramel	9	0	0.0
	Sugar	125	15	0.0
	Sweeteners	67	28	0.0
Special foods	Diet drink mixes (meal replacements)	159	81	0.0
	Diet soup mixes (meal replacements)	6	83	0.0
	Breakfast beverages	125	92	0.1
	Baby foods	424	37	0.0
	Sports/protein powders	607	56	0.0
	Sports gels	26	30	0.0
	Protein and diet bars	392	90	0.1
	Other fitness or diet products	191	74	0.0
	Variety packs not otherwise specified	1	0	0,0
	Milk-based protein drinks	79	55	0.0

Included categories are highlighted.
Fiber intake proportions were adapted from the Australian Bureau of Statistics (ABS) website [1]. The ABS categorization system was mapped to the categorization system used in this analysis to obtain these fiber intake values. We used fiber intake information that related to the entire food supply as there is no information specifically for packaged products.

Supplementary Table S2: Descriptive statistics on nutrients for included products

**Nutrient (per 100g/mL)**	***n***	**Mean**	**SD**	**Min**	**25th quartile**	**Median**	**75th quartile**	**Max**	**Spearman correlation with fiber**
Sugar (g)	21,246	12.0	15.4	0.0	1.9	4.8	16.7	94.0	0.09
Starch (g)	21,246	26.8	23.4	0.0	4.6	21.5	44.9	90.0	0.29
Saturated fat (g)	21,246	3.4	5.3	0.0	0.3	1.2	4.3	66.8	0.32
Unsaturated fat (g)	21,246	8.4	12.0	0.0	0.8	3.3	10.8	67.4	0.47
Protein (g)	21,246	7.4	6.2	0.0	2.6	6.4	10.1	81.1	0.64
Sodium (mg)	21,246	301	517	0	16	225	400	1800	-0.02
Fiber (g)	11,441	5.2	5.9	0.0	1.7	3.6	7.2	90.1	1.00

SD is standard deviation

Supplementary Table S3: Training dataset scores using five-fold cross validation and various combinations of hyperparameters

**Hyperparameters**			**Mean Score using Five-Fold Cross-validation (SD)**			
**Distance metric**	**Weight function**	**Number of neighbors**	**R^2**	**MAE (g per 100 g or 100 mL)**	**ρ**	**CA**
Euclidean	Uniform	1	0.67 (0.06)	1.80 (0.20)	0.79 (0.02)	0.66 (0.02)
Euclidean	Uniform	3	0.74 (0.06)	1.66 (0.21)	0.83 (0.02)	0.67 (0.02)
Euclidean	Uniform	5	0.74 (0.06)	1.66 (0.24)	0.83 (0.02)	0.67 (0.02)
Euclidean	Uniform	8	0.74 (0.06)	1.68 (0.25)	0.83 (0.02)	0.67 (0.02)
Euclidean	Uniform	10	0.73 (0.06)	1.69 (0.25)	0.83 (0.02)	0.67 (0.02)
Euclidean	Distance	1	0.67 (0.06)	1.80 (0.20)	0.79 (0.02)	0.65 (0.02)
Euclidean	Distance	3	0.74 (0.06)	1.61 (0.20)	0.83 (0.02)	0.68 (0.02)
Euclidean	Distance	5	0.75 (0.06)	1.58 (0.20)	0.84 (0.02)	0.69 (0.02)
Euclidean	Distance	8	0.76 (0.06)*	1.58 (0.22)	0.84 (0.02)	0.68 (0.02)
Euclidean	Distance	10	0.76 (0.06)*	1.58 (0.22)	0.84 (0.02)	0.68 (0.02)
Manhattan	Uniform	1	0.67 (0.06)	1.78 (0.20)	0.79 (0.02)	0.66 (0.02)
Manhattan	Uniform	3	0.73 (0.06)	1.66 (0.21)	0.83 (0.02)	0.68 (0.02)
Manhattan	Uniform	5	0.74 (0.06)	1.66 (0.24)	0.83 (0.02)	0.67 (0.02)
Manhattan	Uniform	8	0.74 (0.06)	1.67 (0.26)	0.83 (0.02)	0.68 (0.02)
Manhattan	Uniform	10	0.73 (0.06)	1.71 (0.26)	0.83 (0.02)	0.67 (0.02)
Manhattan	Distance	1	0.67 (0.06)	1.78 (0.20)	0.79 (0.02)	0.66 (0.02)
Manhattan	Distance	3	0.74 (0.06)	1.61 (0.19)	0.83 (0.02)	0.68 (0.02)
Manhattan	Distance	5	0.75 (0.06)	1.58 (0.21)	0.84 (0.02)	0.69 (0.02)
**Manhattan**	**Distance**	**8**	**0.76 (0.06)***	**1.57 (0.22)**	**0.84 (0.02)**	**0.69 (0.02)**
Manhattan	Distance	10	0.75 (0.06)	1.59 (0.23)	0.84 (0.02)	0.69 (0.02)

SD is standard deviation

The Euclidean distance is calculated using $\mathrm{d}(x,y) = \sqrt{\sum_{i=1}^{6} (x_i - y_i)^2}$ and the Manhattan distance is calculated using $d(x,y) = \sum_{i=1}^{6} |x_i - y_i|$, where x_i and y_i refer to the i-th normalized nutrient value of the query point and training point respectively.

The uniform weight function weights neighbors uniformly and the distance weight function weights neighbors by the inverse of their distance.

R^2 is the coefficient of determination. It represents the proportion of variance in fiber content explained by the features in the model, where 1 indicates a perfect fit.

MAE is mean absolute error. It is the average of all absolute errors.

ρ is the Spearman rank correlation. It represents how well the model ranks products from highest to lowest in fiber content, where 1 indicates perfect ranking.

CA is classification accuracy. It measures the proportion of correctly predicted fiber classes. Classes are defined (in g per 100g or 100mL) as: negligible density [0,0.9), low density [0.9, 3.7), medium density [3.7, 7.3), and high density [7.3, 100].

*Three combinations produced an R^2 score of 0.76. To break the tie, MAE was considered

Supplementary Text S1: Example application of the KNN fiber prediction algorithm

Product	Subcategory[1]	Nutrients (per 100g/mL)						d	Fiber (g/100g)
		Sugar (g)	Starch (g)	Protein (g)	Saturated fat (g)	Unsaturated fat (g)	Sodium (mg)		
**Query**	Bars	23.2	41.4	6.8	1.3	4.4	15		
**Neighbor 1**	Bars	21.6	40.4	7.5	0.9	3.9	130	0.05212	11.5
**Neighbor 2**	Bars	18.3	42.1	7.5	1.6	4.8	180	0.08502	12.6
**Neighbor 3**	Bars	20.7	43.2	6.6	1.4	2.9	170	0.08589	11.6
**Neighbor 4**	Bars	22.2	45.8	6.7	1.5	3.4	230	0.08589	8.2
**Neighbor 5**	Bars	20.7	43.6	6.6	1.4	2.9	170	0.08989	12.4
**Neighbor 6**	Bars	23.3	37.7	6.0	0.8	6.5	190	0.09505	9.9
**Neighbor 7**	Bars	14.9	41.1	8.5	1.1	4.3	87	0.10798	10.0
**Neighbor 8**	Bars	22.3	38.9	6.6	4.7	4.6	130	0.10828	9.3

[1]Bars refers to cereal- and nut-based bars

d is the Manhattan distance between the query product and the given neighbor. It is calculated using $d(x,y) = \sum_{i=1}^{6} |x_i - y_i|$, where x_i and y_i refer to the i-th normalized nutrient value of the query point and training point respectively

Estimated fiber content for query product = $\frac{\sum_{j=1}^{8} w_j q_j}{\sum_{j=1}^{8} w_j}$

$$= \frac{\frac{1}{0.05212}\times 11.5+\frac{1}{0.08502}\times 12.6+\frac{1}{0.08589}\times 11.6+\frac{1}{0.08589}\times 8.2+\frac{1}{0.08989}*12.4+\frac{1}{0.09505}\times 9.9+\frac{1}{0.10798}\times 10+\frac{1}{0.10828}\times 9.3}{\frac{1}{0.05212}+\frac{1}{0.08502}+\frac{1}{0.08589}+\frac{1}{0.08589}+\frac{1}{0.08989}+\frac{1}{0.09505}+\frac{1}{0.10798}+\frac{1}{0.10828}}$$

$$= \frac{1019.9728}{94.3758}$$

= 10.8 g per 100g

Supplementary Text S2: Predicting fiber content for example product using ingredients-based algorithm

	Nutrients per 100g or 100mL				Step 2: Predict ingredient proportions (%)	Step 3: Sum the amount of fiber from each ingredient
	**Carbohydrates (g)**	**Protein (g)**	**Total fat (g)**	**Sodium (mg)**		
**Product**	11.0	2.1	5.0	4.9		
**Ingredients**	**Step 1:** Disaggregate ingredients list and determine ingredient nutrient profiles					**Fiber (g per 100g or 100mL)**
Chia seed gel (40%*)						
Filtered water	0.0	0.0	0.0	1.0	29.0	0.0
Chia seed	7.7	16.5	30.7	16.0	11.0	34.4
Apple puree (35%*)	12.7	0.3	0.2	1.0	35.0	2.3
Coconut milk	1.7	1.3	12.8	18.0	12.0	0.2
Apple dice (8%*)	12.7	0.3	0.2	1.0	8.0	2.3
Organic cane sugar	100	0.0	0.0	0.0	4.2	0.0
Lemon juice concentrate	39.6	0.2	0.0	37.0	0.4	0.0
Cinnamon (0.4%*)	25.5	4.2	2.7	18.0	0.4	54.2
						**Total fiber content :** 5g per 100g

*Declared ingredient proportions in the ingredients statement

Step 1:
All ingredient lists in the test dataset were disaggregated. This produced a list of 24,346 unique ingredient names and cleaning of this list (e.g. condensing "strawberry" and "strawberries" to "strawberry") resulted in a list of 744 unique ingredients names in the test dataset. To obtain ingredient-level nutrient profiles, 612 ingredients were mapped to ingredients with nutrient composition data in Food Standard Australia New Zealand's nutrient composition database [2], 25 ingredients were mapped to ingredients with nutrient composition data in FoodSwitch [3], 32 ingredients were mapped to ingredients with nutrient composition data found through google search, and 75 were not mapped as they were assumed to have a value of zero for each nutrient (e.g. colors).

Step 2:
A mathematical optimisation program was used to the predict the proportion of each ingredient. This program considered ingredient-level nutrient profiles to select ingredient proportions that produced a calculated nutrient profile as close as possible to the reported product nutrient profile. This was achieved by minimizing the level of discrepancy between the reported and calculated nutrient profiles as expressed in the objective function (OF):

$$OF = \sum_{i=1}^{4} |r_i - c_i|$$

where r_i is the reported nutrient value of the i-th nutrient (where included nutrients are carbohydrates, protein, total fat, and sodium) and c_i is the value of the i-th nutrient calculated using ingredient weights. All nutrients were standardized so that each nutrient had similar influence in the algorithm.

All predicted ingredient proportions were subject to constraints:

- Any declared ingredient proportions declared in the ingredients list must be used
- Ingredient proportions must be positive
- Ingredient proportions must sum to 100%
- Ingredient proportions must be in descending order

Step 3:
The amount of fiber in each ingredient was summed to determine the overall fiber content in the product

Supplementary Table S4: Confusion matrix on true versus predicted fiber density classes

		True class				
		Negligible	Low	Medium	High	Precision
Predicted class	Negligible	90	57	0	0	0.61
	Low	62	642	114	19	0.77
	Medium	7	180	452	107	0.61
	High	3	44	130	548	0.76
	Recall	0.56	0.70	0.65	0.81	Overall classification accuracy: 71%

Fiber density classes are defined (in g per 100g/mL) as: negligible [0,0.9), low [0.9, 3.7), medium [3.7, 7.3), and high [7.3, 100]. Cells are shaded according to number of products.
Precision measures the proportion of positive classifications that were correct and is calculated using the formula: Precision = true positives/(true positives + false positives)
Recall measures the proportion of actual positives that were classified correctly and is calculated using the formula: Recall = true positives/(true positives + false negatives)

Supplementary Table S5: Comparison approaches on the test dataset

Food category		Performance of the KNN approach				Performance of Ng *et al.*'s approach			
	n	R^2	MAE	ρ	CA	R^2	MAE	ρ	CA
Biscuits	336	0.68	1.57	0.76	0.67	0.30	1.96	0.77	0.61
Bread	379	0.27	1.34	0.62	0.66	-1.69	2.23	0.55	0.53
Breakfast cereals	236	0.59	1.86	0.64	0.82	0.66	1.62	0.66	0.77
Cakes, muffins and pastries	70	0.00	2.82	0.28	0.56	-0.26	2.51	0.57	0.69
Cereal and nut-based bars	167	0.32	1.63	0.57	0.72	-0.49	2.72	0.22	0.48
Crisps and snacks	320	0.26	1.54	0.64	0.64	-0.35	2.34	0.57	0.51
Fruit	174	0.37	2.07	0.69	0.68	0.18	2.47	0.56	0.57
Fruit and vegetable juices	164	0.04	0.37	0.34	0.66	-2.48	0.71	0.29	0.57
Nuts and seeds	100	0.71	1.77	0.62	0.78	0.83	1.55	0.63	0.77
Other cereal and grain products	105	0.95	2.29	0.93	0.76	0.85	4.36	0.87	0.70
Pasta	60	-0.55	1.18	0.36	0.75	-0.35	1.29	0.33	0.80
Ready meals	93	0.23	1.00*	0.29	0.78*	0.53	1.00*	0.44	0.78*
Soup	103	0.89	1.13	0.71	0.75	0.42	2.46	0.65	0.66
Vegetables	148	0.38	1.23	0.68	0.80	0.29	1.54	0.63	0.68
**All**	**2,455**	**0.84**	**1.52**	**0.84**	**0.71**	**0.68**	**2.07**	**0.77**	**0.62**

The superior score for each performance metric is highlighted for each food category
*Wherever KNN and Ng *et al.*'s approach produced the same score for a performance metric, both scores were highlighted
R^2 is the coefficient of determination. It represents the proportion of variance in fiber content explained by the features in the model, where 1 indicates a perfect fit.
MAE is mean absolute error. It is the average of all absolute errors.
ρ is the Spearman rank correlation. It represents how well the model ranks products from highest to lowest in fiber content, where 1 indicates perfect ranking.
CA is classification accuracy. It measures the proportion of correctly predicted fiber classes. Classes are defined (in g per 100g or 100mL) as: negligible density [0,0.9), low density [0.9, 3.7), medium density [3.7, 7.3), and high density [7.3, 100].

References

1. *Australian Bureau of Statistics Australian Health Survey: Nutrition First Results - Foods and Nutrients*. 2011-12.
2. *Food Standard Australia New Zealand. Food Nutrient Database*. 2013; Available from: https://www.foodstandards.gov.au/science/monitoringnutrients/ausnut/foodnutrient/Pages/default.aspx.
3. Dunford, E., et al., *FoodSwitch: A Mobile Phone App to Enable Consumers to Make Healthier Food Choices and Crowdsourcing of National Food Composition Data.* JMIR Mhealth Uhealth, 2014. **2**(3): p. e37.
